# Low-Positive Myelin Oligodendrocyte Glycoprotein (MOG)-Immunoglobulin G (IgG) in Pediatric Demyelinating Disease: How Imaging Features Can Support a MOG Antibody-Associated Disease (MOGAD) Diagnosis

**DOI:** 10.7759/cureus.109271

**Published:** 2026-05-20

**Authors:** Chun Lang Tang, Elaine Wing Shan Fok

**Affiliations:** 1 Radiology and Organ Imaging, United Christian Hospital, Hong Kong, HKG

**Keywords:** acute disseminated encephalomyelitis (adem), flames, magnetic resonance imaging, mogad, mogad‑compatible flair-hyperintense lesions in anti-mog associated encephalitis with seizures, myelin-oligodendrocyte glycoprotein antibody disease, pediatric demyelination

## Abstract

Myelin oligodendrocyte glycoprotein antibody-associated disease (MOGAD) is a rare inflammatory demyelinating disorder that can affect children and remains challenging to diagnose. Neuroimaging plays a key role, as MRI patterns in the brain, optic nerves, and spinal cord differ from those of multiple sclerosis and Aquaporin-4 (AQP4)‑positive neuromyelitis optica spectrum disorder (NMOSD). The 2023 international consensus criteria require a compatible core clinical phenotype, positive serum MOG‑immunoglobulin G (IgG), and exclusion of a better alternative diagnosis, with supportive MRI features when needed.

The authors recently managed a pediatric patient with an acute demyelinating syndrome, positive serum MOG‑IgG, and characteristic compatible lesions. This case illustrates key clinical and imaging features of pediatric MOGAD, shows how the 2023 criteria can be applied in practice, and highlights radiologic clues that help distinguish MOGAD from other demyelinating disorders.

## Introduction

Myelin oligodendrocyte glycoprotein antibody-associated disease (MOGAD) is a rare autoimmune inflammatory demyelinating disorder mainly affecting the central nervous system. It is challenging to diagnose. The 2023 international consensus criteria require a compatible core clinical phenotype, positive serum MOG‑immunoglobulin G (IgG), and exclusion of a better alternative diagnosis, with supportive MRI features when needed [1,2].

Pediatric patients with MOGAD may present with an acute encephalitis-like clinical picture, particularly in cases of cerebral cortical encephalitis with seizures. In such presentations, infectious etiologies remain the most common differential and must be carefully excluded first. These etiologies include viral pathogens, such as herpes simplex virus types 1 and 2, enterovirus, arbovirus, and Japanese encephalitis virus, as well as bacterial infections. MOGAD and other demyelinating diseases are typically considered when an infectious workup is unrevealing and clinical, serological, and radiological features suggest a demyelinating process.

We recently encountered a pediatric patient with an acute demyelinating syndrome, initially low positive serum MOG‑IgG, and typical MRI-compatible features. Our case exemplifies typical lesions in MOGAD‑compatible fluid-attenuated inversion recovery (FLAIR)-hyperintense lesions in anti-MOG-associated encephalitis with seizures (FLAMES), as well as MOGAD-compatible acute disseminated encephalomyelitis (ADEM)‑like presentation in a child, documented against the backdrop of the newly updated 2023 international diagnostic criteria [2]. We demonstrate how to integrate clinical phenotype, serum MOG‑IgG testing, and characteristic MRI features to establish the diagnosis and exclude other differential diagnoses such as multiple sclerosis, Aquaporin-4 immunoglobulin G neuromyelitis optica spectrum disorder (AQP4-IgG+ NMOSD), or MOGAD‑negative ADEM.

This report aims to help raise awareness of the disease, support earlier diagnosis, and ultimately contribute to a more timely initiation of appropriate immunotherapy in similar pediatric patients.

## Case presentation

First episode

An 11-year-old previously healthy boy was admitted for fever, headache, and seizure (status epilepticus) in May 2024. Physical examination showed facial asymmetry with loss of the left nasolabial fold. There was also weakness of the lower left facial muscles with sparing of the forehead muscles. Mild left upper and lower limb weakness was also noted, with the presence of left ankle conus and brisk left lower limb reflexes. His past medical history was unremarkable.

Contrast-enhanced MRI brain showed a unilateral right insular cortex T2-FLAIR hyperintense signal with mild gyral swelling and mild cortical restricted diffusion. T2-FLAIR hyperintense signal was also noted in the right basal ganglia (Figure 1).

**Figure 1:**
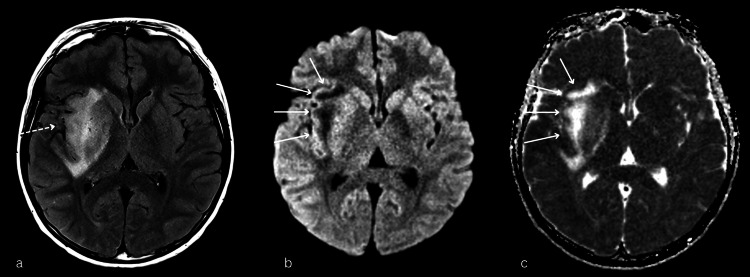
Initial MRI brain with features compatible with FLAMES (a) Initial axial T2-FLAIR MRI of the brain in May 2024 demonstrates a hyperintense swelling of the right insular cortex and basal ganglia (dotted arrow). (b) Axial DWI (b= 1000) and (c) ADC images show corresponding subtle restricted diffusion of the right insular cortex (arrows). Together with clinical features of seizures and fever, these features can be compatible with FLAMES, a sub-entity of MOGAD. Basal ganglia T2-FLAIR hyperintensity is also compatible with basal ganglia encephalitis, which can also be seen in MOGAD. Abbreviations: MRI, Magnetic Resonance Imaging. FLAIR, Fluid-Attenuated Inversion Recovery. MOGAD, Myelin Oligodendrocyte Glycoprotein Antibody-Associated Disease. FLAMES, FLAIR-hyperintense Lesions in Anti-MOG-Associated Encephalitis With Seizures.

Sleep electroencephalogram showed no periodic lateralized epileptiform discharges nor epileptiform activity. Anti-epileptic was prescribed due to status epilepticus as a secondary prophylaxis. All neurological signs and symptoms resolved spontaneously after 1 week, and the patient was discharged a few days later. 

Second episode

In August 2024, the patient developed an episode of headache with bilateral upper and lower limb weakness. MRI brain during this episode showed significant improvement in previous lesions in the right insular cortex and right basal ganglia (Figure 2a). However, multiple new confluent T2-FLAIR hyperintense lesions were noted in bilateral cerebral deep and subcortical white matter, supratentorial cortical gray matter, as well as at the right medulla (Figures 2a, 3a, 4a).

**Figure 2:**
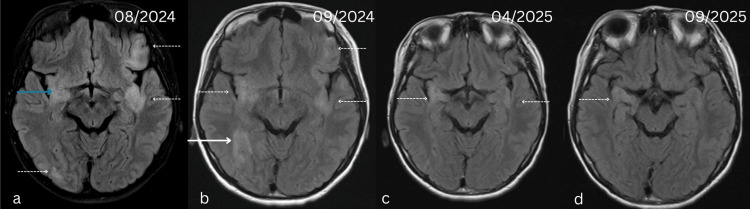
Follow-up MRI brain at the level of the midbrain Follow-up axial T2-FLAIR MRI brain at the level of the midbrain in August 2024 (a) showed that the previous regression of the lesion at the right insular cortex and basal ganglia (solid blue arrow). However, newly noted T2-FLAIR hyperintense lesions were seen at the bilateral supratentorial deep and subcortical white matter and supratentorial gray matter (dotted arrows). Follow-up MRI in September 2024 (b) showed regression of the previous lesion (dotted arrows), but a new lesion was noted at the right parietal and occipital lobe white matter (solid arrow). Follow-up MRI in April 2025 (c) and September 2025 (d) showed regression of all previous lesions. Abbreviations: MRI, Magnetic Resonance Imaging. FLAIR, Fluid-Attenuated Inversion Recovery.

**Figure 3:**
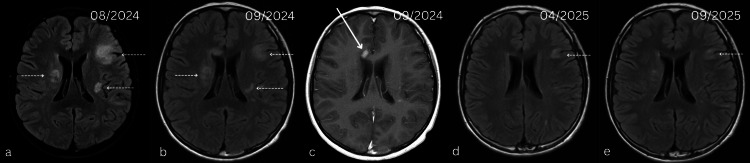
Follow-up MRI brain at the level of the body of the lateral ventricles Follow-up Axial T2-FLAIR MRI brain at the level of the body of the lateral ventricles in August 2024 (a) showed newly noted T2-FLAIR hyperintense lesions at the bilateral supratentorial deep and subcortical white matter as well as supratentorial gray matter (dotted arrows). Follow-up T2-FLAIR MRI in September 2024 showed regression of most lesions (dotted arrows) (b), but a new lesion at the corpus callosum showed nodular enhancement on T1 MPRAGE with contrast (solid arrow) (c). Further follow-up T2-FLAIR MRI in April 2025 (d) and September 2025 (e) showed further resolution of lesions (dotted arrows). Abbreviations: MRI, Magnetic Resonance Imaging. FLAIR, Fluid-Attenuated Inversion Recovery. MPRAGE, Magnetization-Prepared Rapid Gradient-Echo Magnetization-Prepared Rapid Gradient Echo.

**Figure 4:**
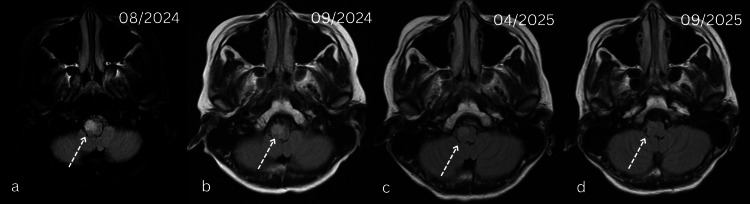
Follow-up MRI brain at the level of the medulla Follow-up axial T2-FLAIR MRI brain at the level of the medulla in August 2024 (a) showed a newly noted T2-FLAIR hyperintense lesion at the right medulla (dotted arrow), which showed regression on follow-up MRI in September 2024 (b), April 2025 (c), and September 2025 (d) (dotted arrow). Abbreviations: MRI, Magnetic Resonance Imaging. FLAIR, Fluid-Attenuated Inversion Recovery.

An ophthalmology examination was performed as the clinical team suspected demyelinating disease. The exam was unremarkable, with no features suggestive of optic neuritis.

Initial serum MOG-IgG testing in this episode using a fixed cell-based assay demonstrated low positive results at a titer of 1:40. Aquaporin-4 (AQP4)-IgG was negative. CSF oligoclonal bands were negative. CSF analysis was also negative for viral PCR, bacterial, fungal, and TB culture.

In view of the clinical suspicion of MOGAD, first-line treatment with high-dose intravenous corticosteroids was recommended in accordance with established practice for inflammatory demyelinating disorders. However, corticosteroid therapy was declined by the patient's parents after detailed discussion. Consequently, a trial of intravenous immunoglobulin (IVIG) was administered over two days as an alternative immunomodulatory treatment. The patient's clinical signs and symptoms improved shortly after.

Third episode

In September 2024, the patient developed another episode of left upper and lower weakness. Repeated MRI brain showed interval reduction in the sizes of previous lesions (Figures 2b, 3b, 4b). However, a new lesion was noted at the right corpus callosum, which showed nodular enhancement (Figure 2c). Another new lesion was also noted at the right parietal and occipital lobe white matter (Figure 3b). MRI spine was normal (not shown).

MRI spectroscopy showed reduced NAA, mildly elevated Cho/Cr ratio (<2), and the presence of a lactate peak. The picture is compatible with neuronal injury accompanied by anaerobic metabolism and mildly increased membrane turnover, indicating an underlying active demyelination process (Figure 5). 

**Figure 5:**
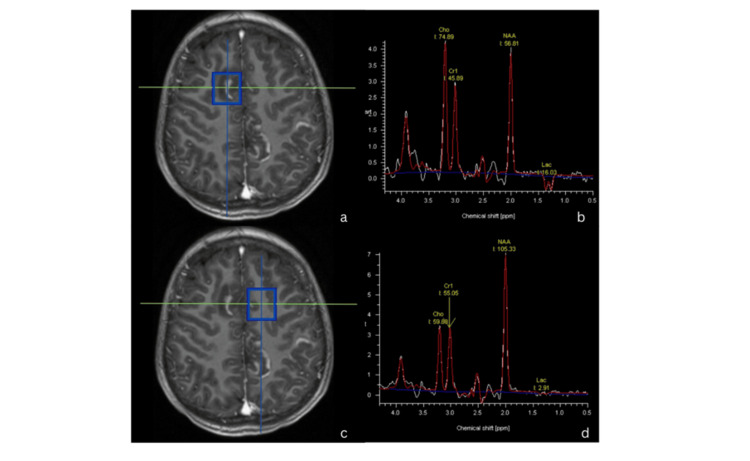
MRI spectroscopy of right frontal lobe subcortical white matter lesion MRI spectroscopy of a right frontal lobe enhancing subcortical white matter lesion showed reduced NAA, mildly elevated Cho/Cr ratio (<2), and the presence of a lactate peak (a, b) compared to normal contralateral white matter (c, d). The picture is compatible with a neuronal injury accompanied by anaerobic metabolism and mildly increased membrane turnover, indicating an underlying active demyelination process. Abbreviations: MRI, Magnetic Resonance Imaging. NAA, N-acetylaspartate. Cho/Cr, Choline/Creatine.

Given the clinical suspicion of MOGAD in the context of an initially low-positive titer, repeat serum MOG-IgG testing was performed during the third clinical episode following discussion with a chemical pathologist. Using a live cell-based assay, MOG-IgG was detected at a higher titer of 1:160, supporting the diagnosis of MOGAD. Signs and symptoms resolved shortly after another two-day course of IVIG. 

Subsequent clinical course

The patient did not experience further clinical signs and symptoms after the three episodes described above. Follow-up MRI studies in April 2025 (Figures 2d, 3c, 4c) and September 2025 (Figures 2e, 3d, 4d) showed resolving lesions at the supratentorial white matter and gray matter, and at the right medulla.

Management and follow-up

After a multi-disciplinary meeting with multiple parties, it was decided that the patient fulfilled the diagnostic criteria for MOGAD, as discussed in the subsequent section. The patient was treated for MOGAD and is currently undergoing maintenance IV immunoglobulin therapy and follow-up at a dedicated pediatric neurology clinic. 

## Discussion

Application of MOGAD diagnostic criteria

Our case is compatible with the waxing and waning course of a demyelinating disease. Further workup with serum analysis was low positive for MOG-IgG in August 2024 and clear positive for MOG-IgG in September 2024. Serum analysis was negative for AQP4-IgG. CSF analysis was unremarkable, and no monoclonal bands were detected.

Looking closer at the latest diagnostic criteria for MOGAD, updated in 2023, all three criteria were fulfilled. The diagnostic criteria require (A) a compatible core clinical phenotype (e.g., optic neuritis, myelitis, ADEM, or cortical/brainstem syndromes), (B) a positive MOG-IgG test based on a cell-based assay, and (C) the absence of a better alternative diagnosis [1,2].

Regarding (B), the criterion of a positive MOG-IgG Test based on a cell-based assay is based on the following: If the MOG-IgG test is clear-positive, no additional supportive features are needed [1,2]; if the MOG-IgG test is low-positive (as in our case in August 2024) or isolated CSF-positive, AQP4-IgG antibody must be negative, 1 or more supportive clinical or MRI features are required to establish the diagnosis [1,2].

We demonstrate how the diagnostic criteria were applied in the following sections.

Criterion A: core clinical demyelinating event

The patient experienced three distinct core clinical demyelinating events, each fulfilling recognized MOGAD clinical phenotypes as defined by the 2023 diagnostic criteria.

First Episode (May 2024): Cerebral Cortical Encephalitis With Seizures

The patient presented with status epilepticus, headache, and fever, accompanied by focal neurological deficits, including facial asymmetry and left-sided weakness. This clinical presentation is consistent with cerebral cortical encephalitis with seizures, a core MOGAD phenotype. 

Second Episode (August 2024): ADEM

The patient developed a headache with bilateral upper and lower limb weakness. This multifocal clinical presentation is consistent with ADEM and cerebral polyfocal deficit, both of which are recognized core MOGAD phenotypes.

Third Episode (September 2024): Cerebral Monofocal Deficit

The patient presented with recurrent left upper and lower limb weakness, representing a cerebral monofocal deficit, another core MOGAD clinical phenotype. 

These three episodes, each corresponding to established core MOGAD phenotypes, satisfy Criterion A.

Criterion B: positive MOG-IgG test by cell-based assay

This case illustrates the analytical limitations of antibody testing in the borderline range. In our case, serum MOG-IgG testing in August 2024 using a fixed cell-based assay demonstrated a low-positive titer of 1:40. Given the clinical suspicion for MOGAD based on characteristic phenotypes across multiple episodes, repeat testing using a live cell-based assay was pursued in September 2024 following discussion with a chemical pathologist. Repeated serum MOG-IgG testing using a live cell-based assay yielded a clearly positive titer of 1:160. This clear positive result definitely satisfies Criterion B.

Prior to the 2023 International MOGAD Panel criteria, a low-positive, fixed, cell-based assay titer frequently led to diagnostic hesitation. The 2023 criteria formally accept a low positive MOG-IgG result when accompanied by one or more supportive clinical or MRI features. Nevertheless, a definitive, live, cell-based assay testing in our case further increased our diagnostic confidence [1-4].

Rationale for Repeat Testing

Both fixed and live cell-based assays have intrinsic limitations that warrant careful consideration when interpreting serum MOG-IgG results. Fixed cell-based assays offer scalability and broad availability, making them suitable for high-throughput testing. However, they may fail to detect MOG-IgG in up to 10-15% of samples that are clearly positive by a live cell-based assay, likely due to loss of conformational MOG epitopes during the fixation process. In contrast, live cell-based assays preserve the native conformation of MOG antigens and typically achieve higher sensitivity and specificity. However, they are technically demanding, requiring flow cytometry or high-resolution fluorescence microscopy, and are therefore primarily available in specialized reference laboratories [3,4].

Given these methodological differences, repeat MOG-IgG testing may be considered in low-positive or weakly positive results with high clinical suspicion. When a fixed cell-based assay yields borderline titers but the clinical and radiological phenotype is characteristic of MOGAD, retesting with a live cell-based assay may reveal higher titers, as demonstrated in our case [3,4].

Interpretation in the Context of Low-Positive Serology and Supportive Imaging Features

Importantly, even if repeat testing had remained low-positive by a live cell-based assay, criterion B in the diagnosis of MOGAD could still be established, provided that supportive MRI features are present alongside negative AQP4-IgG. When initial MOG-IgG testing yielded a low-positive result in August 2024, at least one supportive imaging feature was required to satisfy the diagnostic criteria at that time. Our case demonstrated multiple supportive MRI features characteristic of MOGAD, including a FLAMES pattern with cortical FLAIR hyperintensity and restricted diffusion, basal ganglia involvement with T2 hyperintensity and swelling, confluent poorly marginated ADEM-like lesions in bilateral supratentorial white matter and cortical grey matter, and medullary involvement. The subsequent clear positive result (titer 1:160) on live cell-based assay provides additional diagnostic certainty, but was not strictly required given the strength of clinico-radiological correlation and the presence of multiple supportive imaging features that would have satisfied Criterion B even with persistent low-positive serology [3,4].

First episode (May 2024): Initial MRI showed unilateral right insular cortex T2-FLAIR hyperintensity with gyral swelling and cortical restricted diffusion, features characteristic of FLAMES. Right basal ganglia T2 hyperintensity with swelling, typical of MOGAD-associated basal encephalitis, provided additional supportive imaging evidence (Figure 1).

Second episode (August 2024): Imaging demonstrated interval resolution of prior lesions with multiple new confluent, poorly marginated T2-FLAIR hyperintense lesions involving bilateral supratentorial deep and subcortical white matter, cortical grey matter. These are radiological features highly characteristic of ADEM in the MOGAD spectrum (Figures 2, 3). A brainstem lesion was also noted at the right medulla, which is typical for MOGAD (Figure 4).

Third episode (September 2024): MRI showed new lesions at the right corpus callosum (with nodular enhancement) (Figures 3b, 3c) and right parietal and occipital white matter (Figure 2c), with continued interval reduction of previous lesions (Figures 2, 3). In particular, the lesion with nodular enhancement is more typically seen in ADEM associated with MOGAD than in non-MOGAD-associated ADEM (Figure 3c). 

MR spectroscopy demonstrated reduced NAA, mildly elevated Cho/Cr ratio, and lactate peak, consistent with active demyelination with neuronal injury and anaerobic metabolism (Figure 5).

Criterion C: exclusion of alternative diagnoses

The principal differential diagnoses to exclude are pediatric-onset multiple sclerosis, AQP4-IgG+ NMOSD, and non-MOGAD ADEM.

Multiple sclerosis: The imaging features in our case were not characteristic of MS. The lesions did not involve the calloso-septal interface, did not demonstrate Dawson-finger morphology, and did not show open-ring enhancement. CSF oligoclonal bands were negative, further arguing against MS. The confluent, poorly marginated lesions with interval complete resolution are more typical of MOGAD than MS.

AQP4-IgG+ NMOSD: The lesions did not involve typical NMOSD-associated sites, such as the area postrema or periependymal ventricular surfaces, and did not demonstrate cloud-like enhancement. Serum AQP4-IgG was negative. The cortical involvement with seizures (FLAMES) and basal ganglia encephalitis was also more characteristic of MOGAD rather than NMOSD.

Non-MOGAD ADEM: Clinically, non-MOGAD ADEM is typically monophasic, often following a viral infection or vaccination, whereas MOGAD may be monophasic or polyphasic with relapses. Our patient's relapsing course with three distinct episodes over five months, along with the subsequently clear-positive MOG-IgG serology, strongly favors MOGAD over monophasic non-MOGAD ADEM.

Other alternative causes, such as infectious encephalitides, vasculitis, metabolic or mitochondrial disorders, and neoplastic processes, should also be excluded to establish a diagnosis of MOGAD.

Infectious encephalitis: Infectious encephalitis was considered, given the patient's initial presentation with fever, seizures, and FLAIR hyperintensity involving the cortex and basal ganglia. However, extensive infectious workup in our case, including CSF viral PCR panels and blood cultures, was negative. Furthermore, the relapsing course with interval complete resolution of lesions and emergence of new multifocal lesions in different distributions is inconsistent with typical infectious encephalitis, which generally follows a monophasic course. 

Vasculitis: Central nervous system vasculitis can present with multifocal brain lesions and neurological deficits in children. However, in our case, there was no evidence of vessel wall abnormalities or infarct pattern following vascular territory, arguing against vasculitis.

Metabolic and mitochondrial disorders: A few clinical features argue against a primary metabolic or mitochondrial disorder in our case. First, the complete inter-episode clinical recovery with return to baseline neurological function is atypical for mitochondrial or metabolic disease, which generally shows stepwise deterioration or incomplete recovery between episodes. Second, there was no multi-system involvement, such as developmental delay, cardiomyopathy, or myopathy, to suggest mitochondrial dysfunction. 

Neoplastic processes: In our case, the complete interval resolution of prior lesions with the emergence of new lesions in different locations is inconsistent with neoplastic processes, which typically show progressive growth rather than resolution. 

Etiology and demographics

MOGAD is an autoimmune disease that is characterized by autoantibodies against MOG (a protein found on the surface of myelin). During acute attacks of MOGAD disease, T cells and MOG antibodies cross the blood brain barrier and attack MOG, leading to demyelination [5].

MOGAD is a rare disease with an incidence rate of 0.31 per 100,000 in children and 0.16 per 100,000 in adults. Approximately 30% of MOGAD cases are pediatric patients, in which the median age of onset is 8-9 years old. There is a slight female predominance in MOGAD (around a 1.2:1 female to male ratio) [1,6,7].

Clinical and imaging findings of MOGAD

There are a wide range of clinical presentations for MOGAD, including optic neuritis, transverse myelitis, ADEM, cerebral monofocal or polyfocal deficits, brainstem or cerebellar deficits, or cortical encephalitis [1].

Diagnosing MOGAD is difficult. The 2023 International expert consensus diagnostic criteria for MOGAD offer a standard for better clarification, as discussed previously. There are many imaging features of MOGAD, but characteristic ones for pediatric patients are described in the subsections below. They can occur in isolation or together [7].

In our case, many of the imaging features occurred together. In summary, our case showed typical lesions of FLAMES and basal ganglia encephalitis in the first MRI (Figure 1). In subsequent MRI studies, typical lesions of MOGAD ADEM and a typical brainstem lesion in the medulla were demonstrated.

MOGAD ADEM

In MOGAD ADEM, lesions are typically asymmetrical, bilateral, and multifocal, involving the supratentorial white and/or gray matter, with a predilection for the juxtacortical white matter and deep gray nuclei. Infratentorial white matter is frequently involved as well. The lesions are usually ill-defined, confluent, few in number, but relatively large in size. On MRI, they demonstrate T2 and T2‑FLAIR hyperintensity and may show associated nodular contrast enhancement (in contrast to non-MOGAD-related ADEM) [7,8].

In our case, MRI after the first episode showed compatible multiple confluent, poorly marginated T2/FLAIR hyperintense lesions involving bilateral supratentorial deep and subcortical white matter, cortical gray matter (Figures 2, 3). Moreover, one of the lesions at the right corpus callosum showed nodular enhancement, which is more typical of MOGAD-related ADEM rather than non-MOGAD-related ADEM (Figure 3c).

FLAMES

FLAMES is a clinico-radiological sub-entity of MOGAD. Clinically, it presents with seizure, including or not including headache, fever, or focal neurological deficits related to the cortical lesions. MRI typically shows cortical T2-FLAIR hyperintensity with cortical swelling. Leptomeningeal enhancement and/or restricted diffusion are sometimes observed. The initial MRI of our case showed a typical FLAMES lesion in the initial MRI, with unilateral right insular cortex T2/FLAIR hyperintensity, gyral swelling, and cortical restricted diffusion (Figure 1) [7].

Basal Ganglia Encephalitis

Basal ganglia encephalitis is characterized by T2‑FLAIR‑hyperintense lesions in the thalami and basal ganglia, which can show unclear boundaries. Our case showed a typical basal ganglia encephalitis picture in initial MRI, with right basal ganglia T2 hyperintensity with swelling (Figure 1) [7,9].

Brainstem Lesions

Typical brainstem lesions appear as T2 and T2-FLAIR hyperintense lesions involving the pons, unilateral or bilateral middle cerebellar peduncle, or medulla. Our case demonstrated a typical T2-FLAIR hyperintense lesion involving the right medulla (Figure 4) [7,10]. 

Meningoencephalitis/Aseptic Meningitis

This presents as focal leptomeningeal enhancement, focal T2 hyperintense sulci with no associated brain parenchymal changes. No such imaging findings were seen in our patient [7].

Optic Nerve Lesions

Optic nerve involvement in MOGAD typically presents with bilateral optic neuritis, with T2 hyperintensity and contrast enhancement affecting a long segment of bilateral optic nerve involvement (>50%), typically of the anterior optic nerve. The optic chiasm and tract are typically not involved. Our case did not demonstrate any optic nerve lesions [7,8].

Spinal Cord Lesions

Spinal cord involvement in MOGAD presents clinically and radiologically as transverse myelitis. Transverse myelitis in MOGAD presents as a longitudinally extensive T2 hyperintense lesion involving >3 vertebral segments. On axial T2 images, the lesions are central with predominant gray matter involvement, and may produce a distinctive “H sign". Our case did not demonstrate any spinal cord lesions [7,8].

Treatment and prognosis

Acute Attacks

High-dose IV corticosteroids for three to five days are first-line therapy for acute attacks, followed by an oral taper. IV immunoglobulin (IVIG) and/or plasma exchange are second-line treatments if there is corticosteroid resistance and/or insufficient recovery [11].

Maintenance Therapy

In pediatric MOGAD, maintenance therapy, such as IVIG or oral immunosuppressants (such as azathioprine), is generally recommended for children with relapsing disease or a very severe first attack. Emerging options include IL-6 inhibitors (e.g., satralizumab) and FcRn inhibitors (e.g., rozanolixizumab), with slow steroid tapers (≥3 months) bridging to prevent early relapses [11,12].

Prognosis

Long-term outcome data remain limited. In contrast to other demyelinating disorders, MOGAD tends to have a more favorable prognosis and does not typically exhibit secondary progressive decline [13].

Differential diagnosis of demyelinating disease

The main differentials of MOGAD are other demyelinating diseases. Most common imaging differential considerations include multiple sclerosis, AQP4-IgG-positive NMOSD. These can be differentiated by the pattern of optic nerve involvement, spinal cord involvement, and brain or brainstem involvement [8].

Other differential diagnoses, such as infectious encephalitis, vasculitis, neoplasm, and mitochondrial disease, have been discussed above.

Multiple Sclerosis

Multiple sclerosis can present with relapsing demyelinating events and neurological deficits, but typically shows multiple ovoid periventricular lesions with Dawson-Finger morphology. Calloso-septal interface involvement is typical. Other locations can be the juxtacortical white matter or the deep white matter. Active lesions may show open ring enhancement. Our case demonstrated no typical lesions to suggest multiple sclerosis [8].

Spinal cord lesions are often of short segment, peripherally located, and involve the dorsolateral tracts. Optic nerve involvement is usually unilateral and involves a short segment. CSF oligoclonal bands are more often seen in multiple sclerosis than in MOGAD. In our case, CSF oligoclonal bands were negative, making multiple sclerosis unlikely [8].

AQP4-IgG-Positive NMOSD

AQP4-IgG-positive NMOSD is a key differential. However, it is commonly seen in older populations (around 40 years old) with strong female predominance. Serologically, there is the presence of serum AQP4‑IgG, which was not seen in our case.

In contrast to MOGAD, optic nerve involvement in NMOSD is more often bilateral, involving the optic chiasm, and a long segment of the optic nerves with posterior predominance. Typical cerebral or brainstem lesions are periependymal lesions located around the third and fourth ventricles and the area postrema. Cerebral lesions may also be large lesions involving the subcortical or deep white matter, with cloudlike enhancement. In our case, the lesions do not involve typical locations, making the disease less likely [8].

Like MOGAD, spinal cord lesions also present as long-segment transverse myelitis. However, they tend to have more cord edema and enhancement when compared to MOGAD. Lesions are also more likely to be brighter than CSF on T2-weighted images (“bright spotty lesions”), and have a similar or darker signal intensity on T1-weighted images (“T1 dark lesions”) [8].

ADEM Not Associated With MOG Antibodies or Non-MOGAD ADEM

Monophasic ADEM, unrelated to MOG antibodies, can also produce large, poorly marginated, bilateral T2 hyperintense deep white matter lesions and is a consideration in children with ADEM‑like MRI patterns. ADEM related to MOGAD shares similar morphology but tends to affect juxtacortical white matter and deep gray nuclei. Patchy/nodular parenchymal enhancement is also more commonly seen in ADEM related to MOGAD. Clinically, non-MOGAD ADEM is typically monophasic following infection, whereas ADEM related to MOGAD may be monophasic or polyphasic [7,8,14].

Our case shows a polyphasic presentation. Moreover, the lesions are also poorly marginated and show contrast enhancement, which is more typical for MOGAD ADEM rather than non-MOGAD ADEM [7,8,14].

## Conclusions

MOGAD is a rare and diagnostically challenging entity in the pediatric population, and this case underscores the importance of maintaining a high index of suspicion in children with relapsing or multifocal demyelinating events. In particular, the combination of a compatible clinical syndrome with seizures and focal deficits, FLAMES‑type cortical lesions, ADEM‑like confluent white matter abnormalities, and characteristic brainstem involvement should prompt consideration of MOGAD. Recognizing these imaging patterns and their potential for a waxing and waning course is critical to avoid misclassification as more common demyelinating disorders. Definitive diagnosis, however, requires systematic integration of clinical, serological, and radiological data. Serum testing for MOG‑IgG and AQP4‑IgG should be performed in all suspected cases, with low positive MOG‑IgG interpreted in the context of typical MRI features, negative AQP4‑IgG, and the absence of alternative diagnoses such as pediatric‑onset multiple sclerosis, AQP4‑IgG‑positive NMOSD, or non‑MOGAD ADEM.

Radiologists can play a pivotal role by explicitly describing patterns suggestive of MOGAD such as FLAMES, ADEM‑like lesions, longitudinally extensive transverse myelitis, and bilateral long‑segment anterior optic neuritis. Such an integrated, pattern‑based approach facilitates earlier recognition and appropriate initiation of immunotherapy for affected children and their families.

## References

[1] Trewin BP, Brilot F, Reddel SW, Dale RC, Ramanathan S (2025). MOGAD: a comprehensive review of clinicoradiological features, therapy and outcomes in 4699 patients globally. Autoimmun Rev.

[2] Varley JA, Champsas D, Prossor T (2024). Validation of the 2023 international diagnostic criteria for MOGAD in a selected cohort of adults and children. Neurology.

[3] Budhram A, Flanagan EP (2025). Testing for myelin oligodendrocyte glycoprotein antibodies: who, what, where, when, why, and how. Mult Scler.

[4] Santibáñez T, Valdés JM, Galleguillos L (2026). From low-positive fixed cell-based assay to confirmed myelin oligodendrocyte glycoprotein antibody-associated disease: a paediatric case report. Sclerosis.

[5] Corbali O, Chitnis T (2023). Pathophysiology of myelin oligodendrocyte glycoprotein antibody disease. Front Neurol.

[6] Hor JY, Fujihara K (2023). Epidemiology of myelin oligodendrocyte glycoprotein antibody-associated disease: a review of prevalence and incidence worldwide. Front Neurol.

[7] Pratt LT, Meirson H, Shapira Rootman M, Ben-Sira L, Shiran SI (2025). Radiological features in pediatric myelin oligodendrocyte glycoprotein antibody-associated disease-diagnostic criteria and lesion dynamics. Pediatr Radiol.

[8] Shahriari M, Sotirchos ES, Newsome SD, Yousem DM (2021). MOGAD: how it differs from and resembles other neuroinflammatory disorders. AJR Am J Roentgenol.

[9] Li X, Wu W, Hou C (2023). Pediatric myelin oligodendrocyte glycoprotein antibody-associated disease in southern China: analysis of 93 cases. Front Immunol.

[10] Sechi E, Cacciaguerra L, Chen JJ (2022). Myelin oligodendrocyte glycoprotein antibody-associated disease (MOGAD): a review of clinical and MRI features, diagnosis, and management. Front Neurol.

[11] Whittam DH, Karthikeayan V, Gibbons E (2020). Treatment of MOG antibody associated disorders: results of an international survey. J Neurol.

[12] Wolf AB, Palace J, Bennett JL (2023). Emerging principles for treating myelin oligodendrocyte glycoprotein antibody-associated disease (MOGAD). Curr Treat Options Neurol.

[13] Lopez-Chiriboga AS, Sechi E, Buciuc M, Chen JJ, Pittock SJ, Lucchinetti CF, Flanagan EP (2020). Long-term outcomes in patients with myelin oligodendrocyte glycoprotein immunoglobulin G-associated disorder. JAMA Neurol.

[14] Fan X, Li Q, Li T (2022). Radiological features for outcomes of MOGAD in children: a cohort in southwest China. Neuropsychiatr Dis Treat.

